# Quantitative and Qualitative Assessment of Adhesive Thrombo-Fibrotic Lead Encapsulations (TFLE) of Pacemaker and ICD Leads in Arrhythmia Patients—A *Post Mortem* Study

**DOI:** 10.3389/fcvm.2020.602179

**Published:** 2020-11-30

**Authors:** Jonas Keiler, Marko Schulze, Ronja Dreger, Armin Springer, Alper Öner, Andreas Wree

**Affiliations:** ^1^Department of Anatomy, Rostock University Medical Center, Rostock, Germany; ^2^Divisions of Cardiology, Rostock University Medical Center, Rostock, Germany; ^3^Medical Biology and Electron Microscopy Center, Rostock University Medical Center, Rostock, Germany

**Keywords:** foreign body reactions, vascular fibrosis, neointimal formation, vascular adhesion, thrombus organization

## Abstract

The demand for cardiac implantable electronic devices for arrhythmia therapy is still unabated and rising. Despite onward optimizations, lead-related problems such as infections or fractures often necessitate lead extraction. Due to adhesive thrombo-fibrotic lead encapsulations (TFLE) transvenous lead extraction is challenging and risky. However, knowledge on TFLEs and possible correlations with technical lead parameters and dwelling time (DT) were hitherto insufficiently studied. Therefore, we analyzed TFLEs of 62 lead from 35 body donor corpses to gain information for a potential lead design optimization. We examined both TFLE topography on the basis on anatomical landmarks and histo-morphological TFLE characteristics by means of histological paraffin sections and scanning electron microscopy of decellularized samples. The macroscopic analysis revealed that all leads were affected by TFLEs, mainly in the lead bearing veins. Half (47.2%) of the right-ventricular leads possessed adhesions to the tricuspid valve. On average, 49.9 ± 21.8% of the intravascular lead length was covered by TFLE of which 82.8 ± 16.2% were adhesive wall bindings (WB). The discrete TFLEs with at least one WB portion had a mean length of 95.0 ± 64.3 mm and a maximum of 200 mm. Neither sex, DT nor certain technical lead parameters showed distinct tendencies to promote or prevent TFLE. TFLE formation seems to start early in the first 1–2 weeks after implantation. The degree of fibrotization of the TFLE, starting with a thrombus, was reflected by the amount of compacted collagenous fibers and likewise largely independent from DT. TFLE thickness often reached several hundred micrometers. Calcifications were occasionally seen and appeared irregularly along the TFLE sheath. Leadless pacemaker systems have the advantage to overcome the problem with TFLEs but hold their own specific risks and limitations which are not fully known yet.

## Introduction

With approximately one million devices per year artificial pacemakers are the most frequently applied implants in humans worldwide (1). In the next years, the demand for cardiac implantable electronic devices (CIEDs) including pacemakers and implantable cardiac defibrillators (ICD) for arrhythmia therapies will further increase due to the demographic transformation with aging societies (2). Although CIED implantations are routine procedures with generally low complication rates, various issues, including implant-related inflammatory infections and system failures, can appear in the follow-up (3).

Transvenous lead extraction (TLE) is usually indicated in the case of lead-related issues such as fractures or infections (3–5). However, adhesive thrombo-fibrotic lead encapsulations (TFLE) which are common and appear even after a short dwell time (DT) complicate simple traction and require special retrieval techniques based on mechanical rotating (6, 7), electrosurgical dissection (8) or laser cutting (9, 10). As a consequence, TLE is a challenging and risky procedure often resulting in the physician's decision to abandon the lead in the patient (3, 11–13).

These shortcomings with conventional CIEDs could be overcome with leadless systems which are on the rise (14–17). However, leadless CIEDs are hitherto limited to bradycardia treatment and do not cover defibrillation function. Therefore, artificial permanent pacemakers and implantable cardioverter defibrillators with up to three transvenous leads are still preferred systems for the treatment of cardiac arrhythmias, making TFLEs still a major challenge in case of indicated lead extraction.

Although numerous studies on TFLEs after clinical lead extraction, in corpses and animal models exist (18–34) most previous investigations assessed topography and histo-morphology of TFLEs in humans only superficially. Proper quantitative analyses of TFLE frequencies affecting the various vascular areas are widely lacking. To complement data from previous studies in more detail and to gain information for the optimization of CIEDs in terms of reduced TFLE and better extractability, we re-evaluated the topographic and histological patterns of TFLEs in arrhythmia patients with CIEDs *post mortem*. Possible correlations between TFLE characteristics and lead properties and implant duration are discussed.

## Materials and Methods

TFLEs of permanent CIED leads (*n* = 62) were analyzed systematically in 35 hearts from body donor corpses. Quantitative parameters such as length and position of the encapsulations relative to the distal lead tip and qualitative histo-morphological characteristics for 60 leads (34 heart) were assessed and comparatively mapped including the topography of individual anatomical landmarks. Left ventricular (LV) leads were studied only proximally to the ostial opening of the coronary sinus.

This study was approved by the local ethics committee (vote A2015-0141).

### Dissection and Fixation

Lead-bearing hearts and veins (cephalic vein, subclavian vein, brachiocephalic vein, upper caval vein) were dissected *in toto* from corpses carrying an CIED (57.1% males; mean age 84 ± 7.7 years; range: 63–97 years). Hearts and vessels were either dissected during anatomical classes from previously preserved human corpses (perfusion and immersion with 2% formaldehyde in 58% aqueous ethanol) or taken from fresh corpses with subsequent tissue fixation (immersion in 3.7% phosphate-buffered paraformaldehyde). After dissection and fixation, all hearts were stored in 70% aqueous ethanol.

### Macroscopic Analysis

Lead bearing veins and heart chambers were carefully opened by means of a cut along the vascular axis and fenestration of the cardiac wall, respectively. TFLEs were documented by photography with a Canon EOS 600D and a topographic mapping using technical lead components such as lead tip (cathode), ring electrode (anode), ICD shock coils and anatomical landmarks as references. Lengths were measured using a laboratory straightedge (Shinwa, Tsubame, Japan).

For the TFLEs, distinction was made between encapsulations which were adhesive (wall binding; WB) and non-adhesive (free sheath; FRS) to the adjacent cardiovascular tissue. Moreover, it was noticed if a TFLE affected two or more leads at a certain position (lead-to-lead-binding; LLB).

### Microscopic Analysis

Due to the large number and length of present TFLEs in our study population we limited our microscopic analysis to 21 randomly selected leads (DT: 0.5–250 months), and analyzed 73 TFLE loci histologically to assess their tissue composition. TFLEs were dissected by first cutting through the lead distally and proximally to the region of interest with strong dissection scissors followed by removal of the metallic inner lead core (wires) by means of careful traction. Samples with macroscopic calcification were de-calcified in aqueous EDTA for 2–3 weeks. The tissue samples were then dehydrated via a series of increasing concentrations of ethanol, followed by substitution with xylene and finally embedded in paraffin. With a microtome (LEICA RM2255, Wetzlar, Germany) 5 μm thin serial sections were cut. Deparaffinized histological sections were stained with Heidenhain's Azan trichrome and HE (hematoxyline and eosin). Microphotographs of histological sections were taken with the bright field microscopes DM6 (Leica, Wetzlar, Germany) and Axio Imager M2 (Zeiss, Oberkochen, Germany). Maximum TFLE thickness was measured using Fiji, the image processing-package of ImageJ (https://imagej.net/Fiji).

Referring to a previous study on the thrombo-fibrotic pathway in thrombo-embolic pulmonary hypertension (35), our histological findings were classified by means of a simplified 3-stage scoring system which reflects the degree of the fibrotic reaction of the studied tissue:

#1 – *thrombotic capsule*. Fibrin rich late thrombus/early organized thrombus (granular-thrombotic) with amorphic texture, variable cellularity (granulation tissue), erythrocyte deposition and low collagen content throughout the encapsulation (Figure 1A).
#2 – TFLE with mixed tissue representing a late organized thrombus with intermediate collagen content (Figure 1B).
#3 – TFLE with densely packed collagen fibers and low cellularity (Figure 1C).

**Figure 1:**
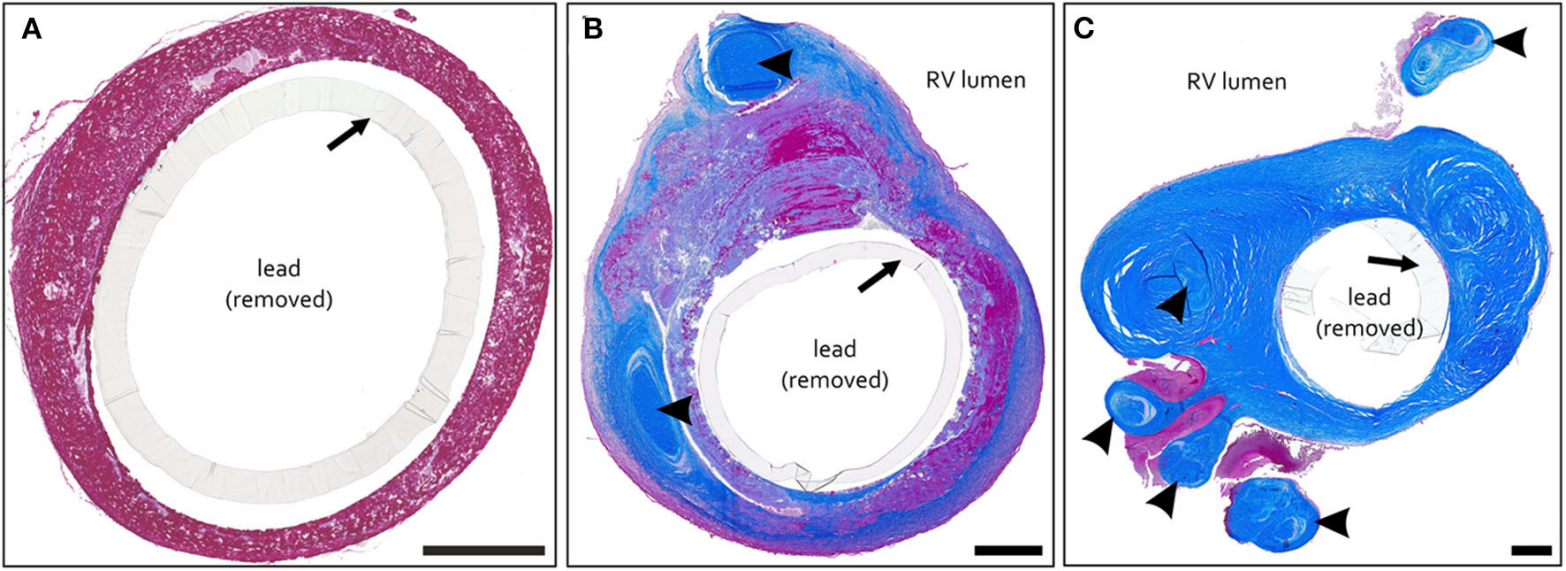
**(A–C)** Exemplary paraffin sections of thrombo-fibrotic lead encapsulations (TFLEs) stained with Azan trichrome stain representing different degrees of TFLE. The inner metallic lead wires were removed before sectioning. Arrows indicate remained outer lead insulation. **(A)** TFLE score #1 – thrombotic encapsulation in the right ventricle (RV) after 28 months dwell time (DT). **(B)** TFLE score #2 – mixed tissue in the RV at the level of the tricuspid valve after 15 months DT (arrowheads indicate wall binding with tendons). **(C)** TFLE score #3 – densely packed collagenous capsule in the RV at the level of the tricuspid valve after 61 months DT. Tendons (arrowheads) are fully or partially ingrown by the TFLE. Scale bar = 500 μm.

Additionally, decellularized tissue samples of seven TFLE-loci from three leads were processed for scanning electron microcopy (SEM) for examination of the collagenous TFLE fiber structure. For this purpose, exemplary TFLE tissue samples were washed in PBS and distilled water and subsequently treated with sodium hydroxide following a modified protocol by Ohtani (36). After maceration for 5 days at room temperature, samples were washed for 1 d in distilled water with the addition of 0.01% thiomersal and 0.2% NaN_3_, followed by fiber stabilization with aqueous 1% tannic acid for 2 h. After washing in distilled water + thiomersal/NaN_3_, samples were dehydrated via a series of increasing concentrations of ethanol, followed by conductive staining in 0.3% ethanolic phosphotungstic acid for 1 d. Samples were than washed in absolute ethanol and transferred to 100% acetone before critical-point-drying (EMITECH KPT 850, Ashford, UK). Immediately after CPD the samples were mounted on Aluminum SEM-carrier with adhesive conductive carbon tape (Plano, Wetzlar) and coated with carbon under vacuum (EM SCD 500, Leica). Finally samples were analyzed by a field emission scanning electron microscope (Merlin VP Compact, Zeiss).

### Assessment of DT and Technical Parameters

Of the 62 studied leads, 23 were right atrial (RA), 36 were right ventricular (RV) and 3 were left ventricular (LV) leads. Of the RVleads, 11 were shock coil leads of an ICD. LV leads for cardiac resynchronization therapy (CRT) were studied only proximally to the ostial opening of the coronary sinus. Taken together, two 3-chamber systems (CRT), 22 dual-chamber pacemakers (with 1 biventricular CRT-system) and 11 single chamber pacemakers were studied. Besides four exceptions, implanted device and leads (Table 1) were from the same manufacturer in the individual corpses.

**Table 1:** Number of implanted devices (*n* = 35) and studied leads (*n* = 62) sorted by manufacturers.

	**Medtronic**	**Biotronik**	**SJM**	**Viatron**	**Sorin**	**Boston Scientific**	**Unknown**
Devices	16	13	5	–	1	–	–
Leads	28	22	4	4	2	1	1

DT and technical parameters such as diameter and outer insulation (Table 2) of the studied leads were assessed by device read out and manufacturers [Abott Laboratories (St. Jude Medical), Biotronik, Medtronic] request by means of the anonymized individual device code. DT determination was possible for 54 leads (87.1%) with limited information for two older leads for which only the minimum DT (60 and 240 months) was assessable. Mean dwell time of all CIED leads was 74.1 ± 56.8 months (min: 2 weeks, max: 20 years).

**Table 2:** General characteristics of the studied leads (*n* = 62).

Mean lead diameter	2.1 ± 0.4 mm
Smallest lead diameter	1.2 mm
Largest lead diameter	2.8 mm
Polyurethane insulation (PU)	*n* = 22
Silicone insulation (Si)	*n* = 35
Co-polymer (PU + Si)	*n* = 4

*Technical parameters were not available for one abandoned ICD lead which was without lead number and device entry*.

Fisher's exact test (significance for *p* < 0.05) and regression test were used for statistical analyses.

## Results

### Macroscopic Analysis

**Figure 2:**
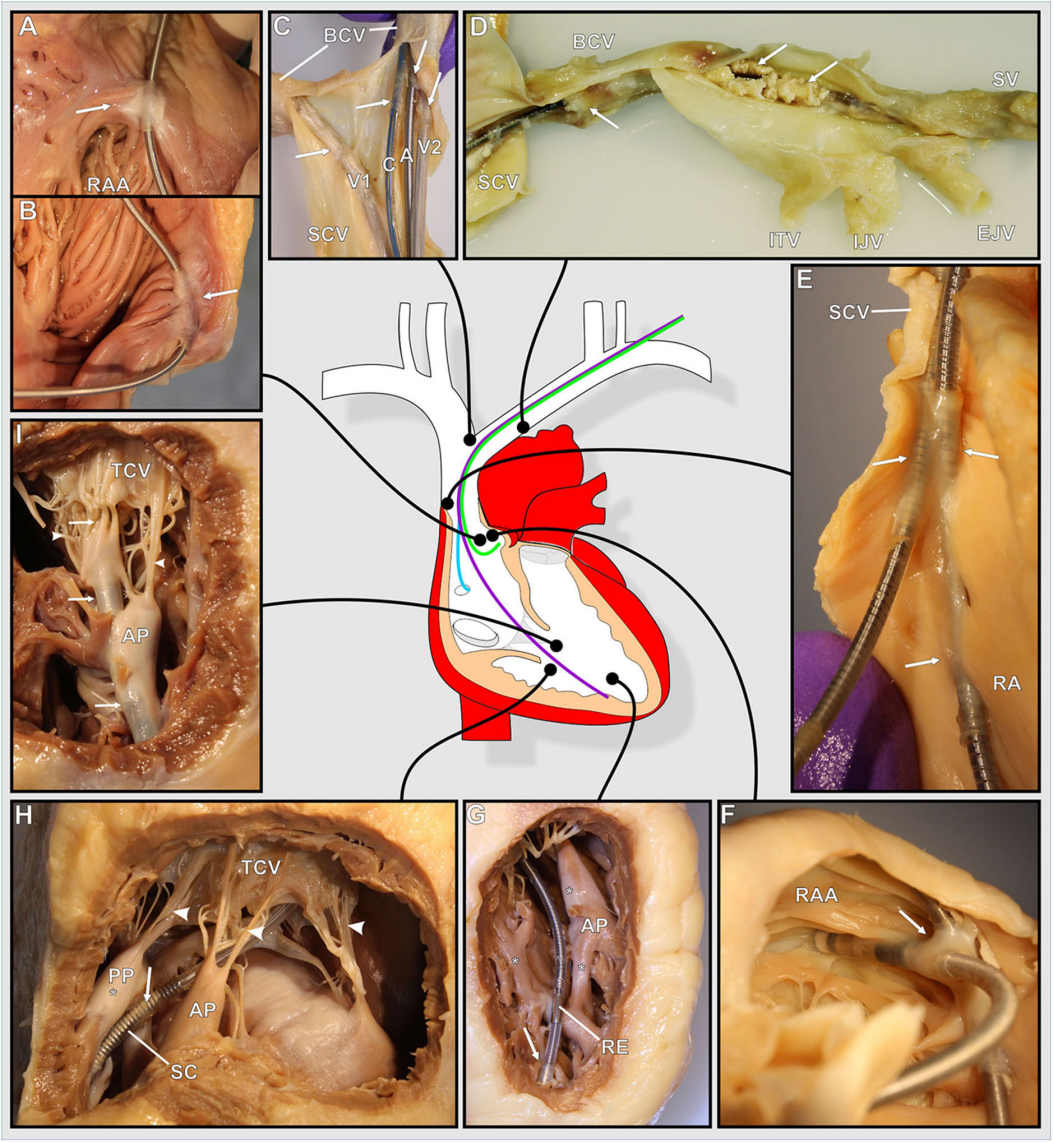
**(A–I)** Photographs of various exemplary thrombo-fibrotic lead encapsulations (TFLEs) with a schematic illustration of the human heart showing the anatomical sites of the TFLEs in veins, right atrium (RA), and right ventricle (RV). Arrows indicate TFLE loci. Arrowheads indicate the tendinous chords (TC) of the tricuspid valve (TCV). **(A,B)** TFLE at the crista terminalis in the RA (DT: 60 months). **(C)** Bifurcation of the superior caval vein (SCV) with 4 leads (A – RA lead, DT: 120 months; C – left ventricular lead, DT: 3 months; V1 – abandoned RV lead in the contralateral brachiocephalic vein (BCV), DT: ~240 months; V2 – RV lead, DT: 240 months). **(D)** Strongly calcified TFLE in the veins (DT: 84 months). **(E)** Lead-to-lead binding at the cavoatrial junction (DT: 80 months). **(F)** Lead-to-lead binding in the RA (DT: 114 months). **(G)** Short TFLE at the ventricular lead tip. Whitish fibrotic lesions (asterisks) at papillary muscle (AP) and trabeculas adjacent to the lead (DT: 64 months). **(H)** Thin TFLE sheathing the ventricular shock coil of an ICD lead. Posterior papillary muscle (PP) with whitish fibrotic lesion (asterisk) adjacent to the SC (DT: 145 months). **(I)** TFLE adhesive to papillary muscles, TC and TCV in the RV (DT: 145 months). A, atrial lead; AP, anterior papillary muscle; BCV, brachiocephalic vein; C, left ventricular lead (CRT); EJV, external jugular vein; IJV, internal jugular vein; ITV, inferior thyroid vein; PA, posterior papillary muscle; RAA, right auricular appendage; RE, ring electrode (anode); SCV, superior caval vein; SV, subclavian vein.

**Figure 3:**
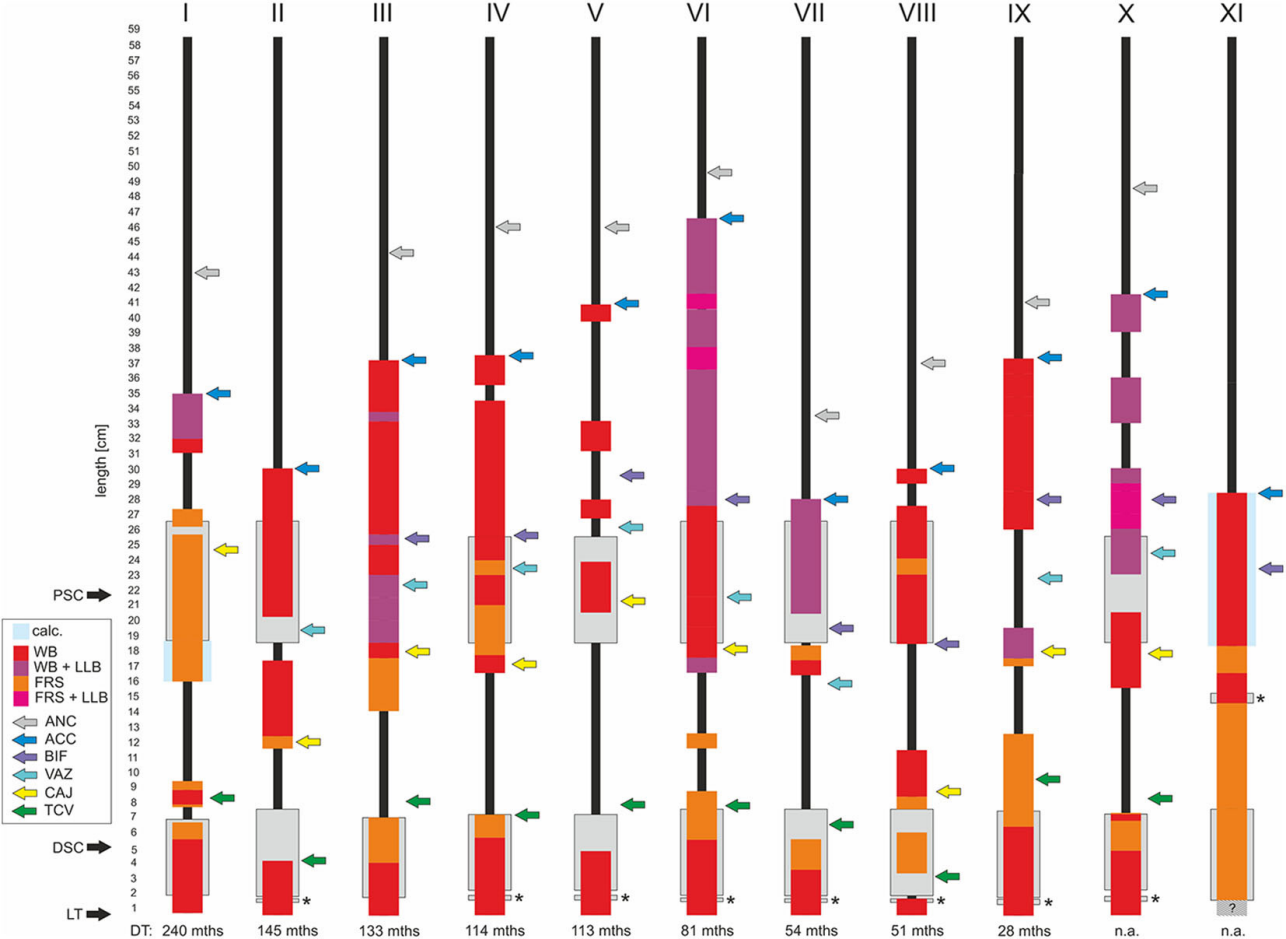
Schematic illustration of thrombo-fibrotic lead encapsulations (TFLEs) of right ventricular leads of implantable cardioverter-defibrillators (ICD) from different corpses (I-XI). Sorted by dwell time (DT). Position and length of TFLEs were measured relative to the distal lead tip (LT). The type of TFLE is illustrated by means of different color codes. Red: adhesion to adjacent vascular or cardiac wall (wall binding = WB); purple: WB + adhesion to adjacent lead (lead-to-lead-binding = LLB); orange: encapsulation without adhesion to adjacent vascular or cardiac wall (free sheath = FRS); pink: FRS + LLB. Anatomical landmarks are indicated by colored arrows. Green = tricuspid valve (TCV); yellow = cavoatrial junction (CAJ); violet = brachiocephalic confluence (upper caval vein bifurcation = BIF); light blue = azygos vein (VAZ); dark blue = lead entry into vein (access = ACC); gray = anchoring sleeve of lead (ANC). DT is given in months (mths). DSC, distal shock coil; PSC, proximal shock coil. Asterisks indicate ring electrode (anode) in bipolar leads. Hatched area in XI indicates unclear situation due to hidden portion. Lead types and technical parameters are given in Supplementary Table 1.

Despite varying prevalences, TFLEs were detected at virtually all anatomical regions adjacent to the course of the implanted lead (Figure 2). Lead bearing veins (cephalic vein, subclavian vein, brachiocephalic vein, superior caval vein) and the cavoatrial junction were mostly affected by TFLEs. Adhesions to the atrioventricular tricuspid valve (TCV) cusps or their tendons were present only occasionally. On average, half (49.9 ± 21.8%) of the intravascular lead length was covered by TFLE of which 82.8 ± 16.2% were wall bindings (WB) adhesive to adjacent vascular tissue. Along their length, up to five separate sheaths covered each lead (mean 2.8 ± 1.1). The discrete TFLEs with at least one WB portion had a mean length of 95.0 ± 64.3 mm. The longest continuous WB was found on the upper portion of an ICD lead with a dwelling time (DT) of 81 months and had a length of 200 mm (Figure 3). Differences in TFLEs between males and females were only minor. Taken together, all leads were affected by WB at one or another position. Intracardiac structures (RA, RV, and/or TCV) were affected by WB in 62.9% of all studied leads, with RV leads having a higher portion of 87.1%. The lead bearing veins were affected most by WB with 98.3% (Table 3).

**Table 3:** Selected anatomical loci with prevalences of adhesive thrombo-fibrotic lead encapsulations (wall binding – WB).

**Wall binding**	**Prevalence**
Intravascular WB (*n* = 60)	100.0%
@ lead bearing veins (*n* = 60)	98.3%
@ brachiocephalic vein (*n* = 61)	90.2%
@ superior caval vein (*n* = 59)	74.6%
@ cavoatrial junction (*n* = 56)	57.1%
@ tricuspid valve (RV-leads; *n* = 36)	47.2%

*Number of studied samples for each anatomical loci in brackets varied due to different degrees of preservation. RV, right ventricle*.

**Figure 4:**
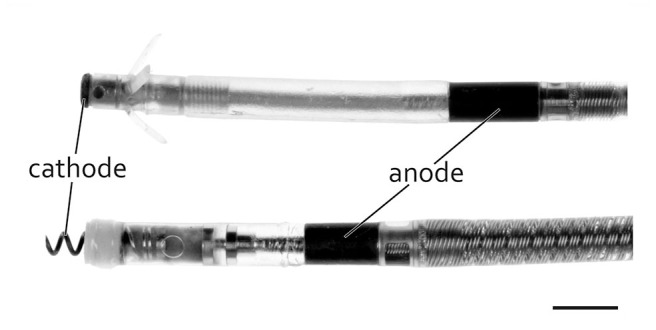
Typical bipolar pacing leads with distal tip (cathode) and ring (anode) and with passive (top) and active (bottom) fixation. Scale bar = 3 mm.

**Figure 5:**
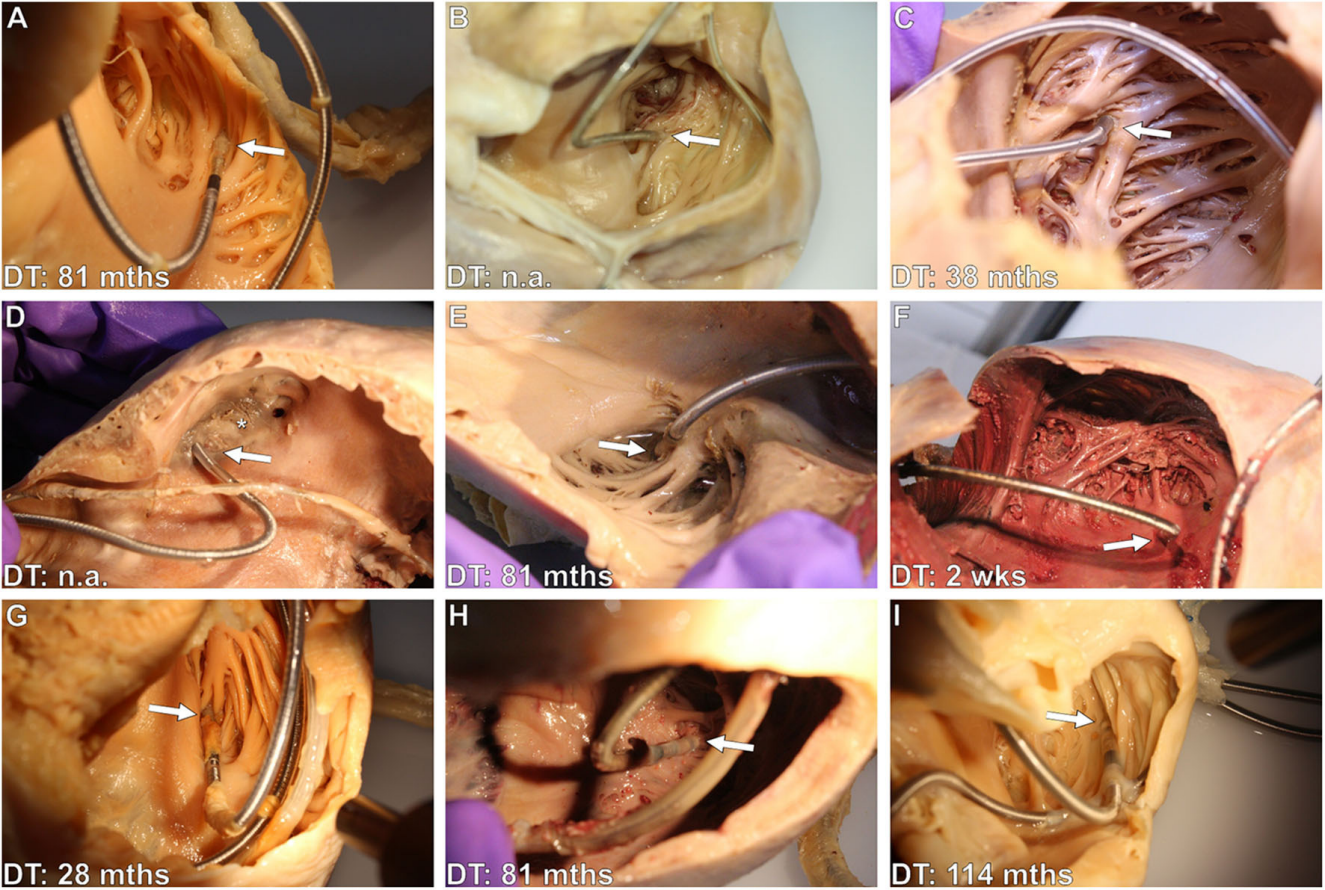
**(A–I)** Exemplary photographs of various right atrial (RA) lead tips with varying degrees of thrombo-fibrotic lead encapsulations (TFLEs) in hearts of different body donor. Arrows point at the lead tip (cathode region) at the transition to the right auricular appendage (RAA) or between the pectinate muscles of the RAA. Distal lead ingrowth (i.e., of the distal 5mm) is seen in **(A,D–I)**. RA leads in **(B)** (unipolar) and **(D)** (bipolar) were passively fixed with anchor tines, the remaining (bipolar, **A,C,E–I**) were actively fixed with a screw. Asterisks indicates old thrombic material filling the RAA. DT, dwell time; mths, months; wks, weeks.

**Figure 6:**
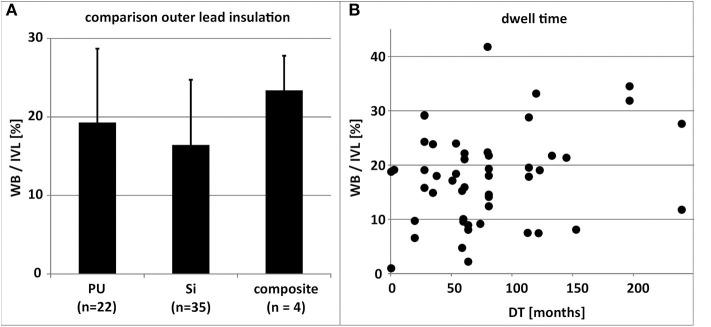
**(A)** Comparison of the mean percentual adhesive thrombo-fibrotic lead encapsulation (TFLE) according to the material of the outer lead insulation (*n* = 49). Error bars = standard deviation. **(B)** Depiction of non-significant relation (linear regression; R^2^ = 0.0631) between lead dwell time (DT) and percentual adhesive TFLE (*n* = 49). Composite, Si+PU, IVL, intravascular lead length; PU, polyurethane; Si, silicone; WB, wall binding length.

The type of distal lead fixation (Figure 4) via anchor tines (“passive”) or screw (“active”) did not have any distinguishable effect on the degree (length and thickness) of TFLE on the distal portion of the lead. Active fixation was present in 42.9% of the RV leads and 82.6% of the RA leads. Although a distal steroid releasing system (present in 93.4%) may have reduced local inflammatory processes immediately after implantation (e.g., via dexamethasone) distal lead ingrowth (i.e., of the distal 5 mm) was observed in the case of 66.7% of RA leads (Figure 5) and 93.5% of RV leads. At least 50% of the ring electrode (anode) surface was covered by TFLE in 68.8% of the RA leads (Figures 5F,H,I) and 87.0% of the RV leads. Considering the electrode coating, a covering was present in 66.7% for iridium (12/18), 58.8% for platinum (10/17), and 50.0% for titanium nitride (3/6) and platinized iridium (1/2). We found no clear trend that the degree of coverage (<50 vs. ≥50%) correlates with the type of surface material of the ring electrode (*p* = 0.73 for platinum vs. iridium with Fisher's exact test). No differences or trends in the degree of TFLE were detected between different materials (silicone, polyurethane, or silicone-polyurethane-co-polymer) of the outer lead insulation (Figure 6A).

Despite silicone backfill of the proximal and distal shock coils (platinum or platinized iridium surface) we observed partial or full coverage of the shock coils with TFLE (Figures 2H,I) in all ICD leads (*n* = 11) with an average DT of 10 years (119.9 ± 73.6 months; minimum DT = 28 months) (Figure 3).

Generally, the lead dwelling time (DT) had only limited effect on TFLE length (*R*^2^ = 0.0631; Figure 6B).

The few portions of the lead which were not in direct contact with the venous endothelium or endocardium—primarily those in the RA—remained free of fibrotic adhesions to the vascular or cardiac wall. Nonetheless, TFLEs without adhesions to adjacent tissue (“free sheaths”) or with adhesion to adjacent leads (“lead-to-lead-bindings”) were occasionally detected in the RA.

### Microscopic Analysis

#### Histology

**Figure 7:**
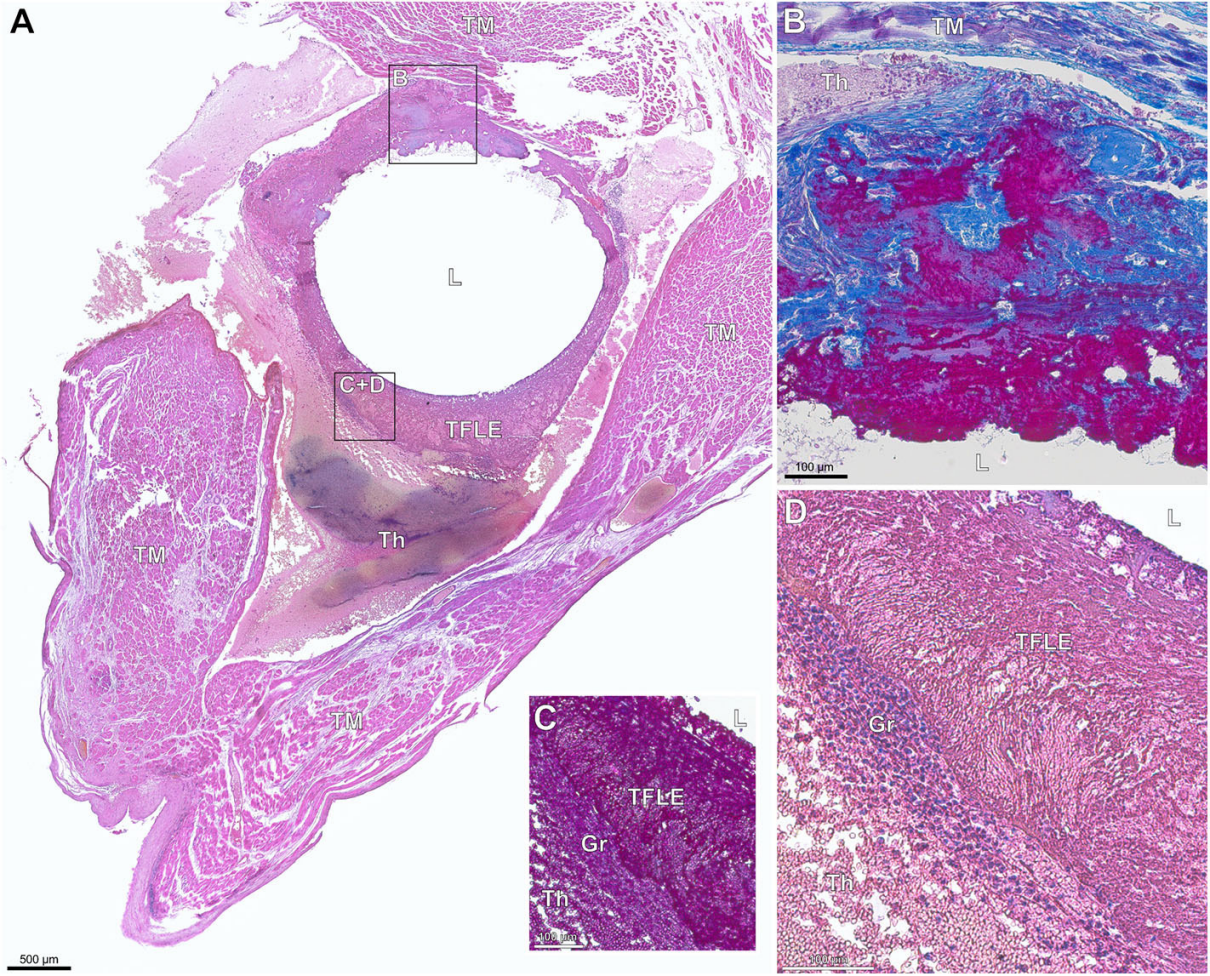
**(A–D)** Paraffin sections of early thrombo-fibrotic lead encapsulation (TFLE) between the trabecular muscles of the right ventricle stained with hematoxylin-eosin (HE) or Azan trichrome. Male, 87 years. Dwelling time: 2 weeks. **(A)** Overview. HE staining. **(B)** Magnified TFLE region adjacent to trabecular muscle with early fibrotization through deposition of collagen fibers (blue) within granular-thrombotic tissue. Azan staining. **(C,D)** Magnified TFLE region with capsule formed by amorphous late organized thrombus tissue with adjacent granulation tissue in the outer capsule zone. Azan **(C)** and HE **(D)** staining. Gr, granulation tissue; L, lead (removed); Th, fresh thrombus; TM, trabecular muscles.

**Figure 8:**
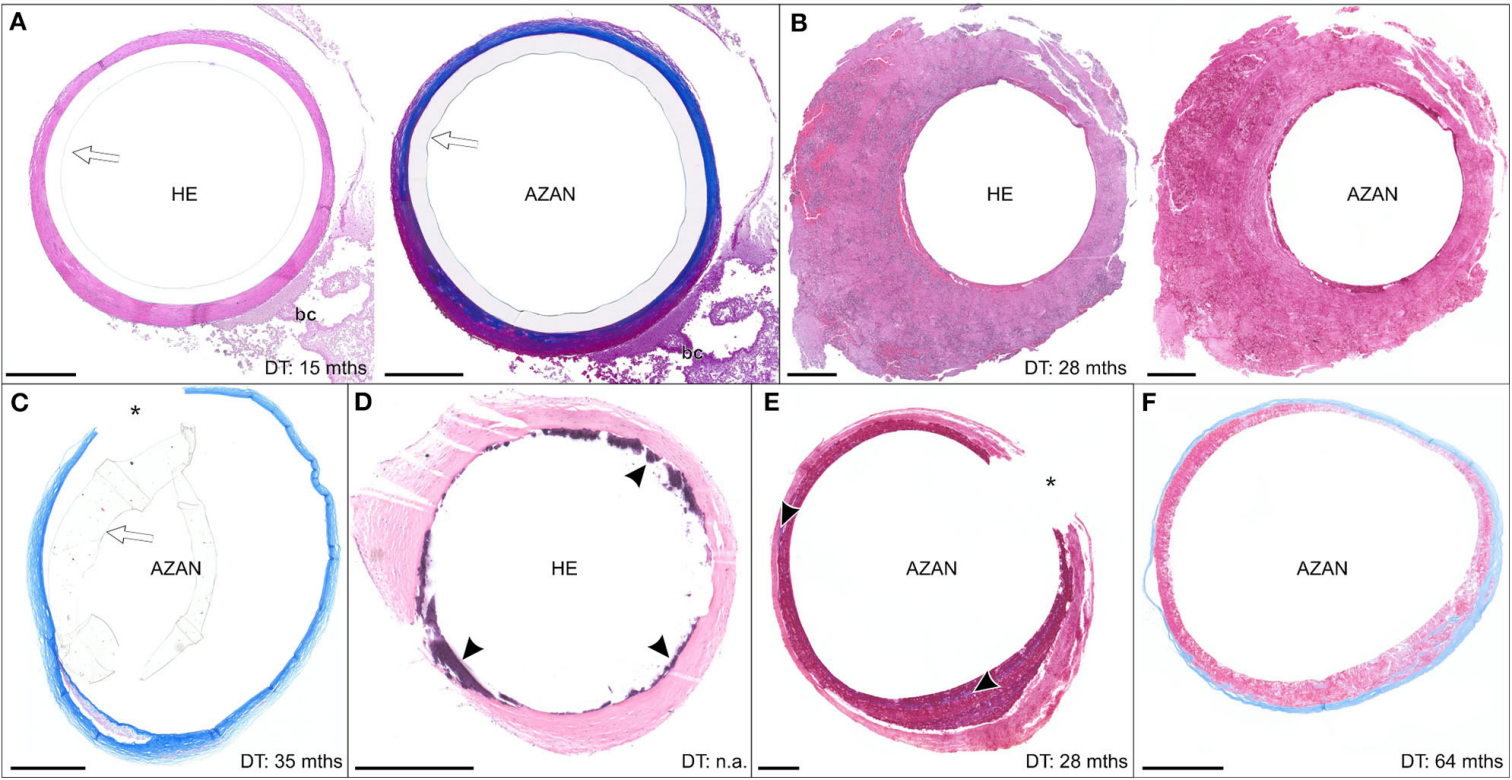
**(A–F)** Exemplary paraffin sections of thrombo-fibrotic lead encapsulations (TFLEs), stained with hematoxylin-eosin (HE) or Azan trichrome, representing free sheaths (FRS) without wall binding. Depicted sections represent various degrees of TFLE, dwelling times (DT) and anatomic locations. The inner metallic lead wires were removed before sectioning. Arrows indicated remained outer lead insulation. Asterisks indicate artificially ruptured TFLE. **(A)** TFLE from the right ventricle (RV) with dense collagenous inner and granular-thrombotic outer layer. **(B)** Thick granular-thrombotic TFLE from the RV. **(C)** TFLE with loose collagenous fibers at the level of the tricuspid valve (TCV). Note the prominent granular-thrombotic inclusion. **(D)** TFLE from the RV with inner calcareous plaques (arrowheads). **(E)** TFLE at the level of the TCV with 2 layers of different granular-thrombotic tissue. The inner layer exhibits signs of fibrotization (arrowheads). **(F)** TFLE from the superior caval vein with inner loose granular-thrombotic and outer collagenous layer. Bc, blood cells. Scale bars = 500 μm.

**Figure 9:**
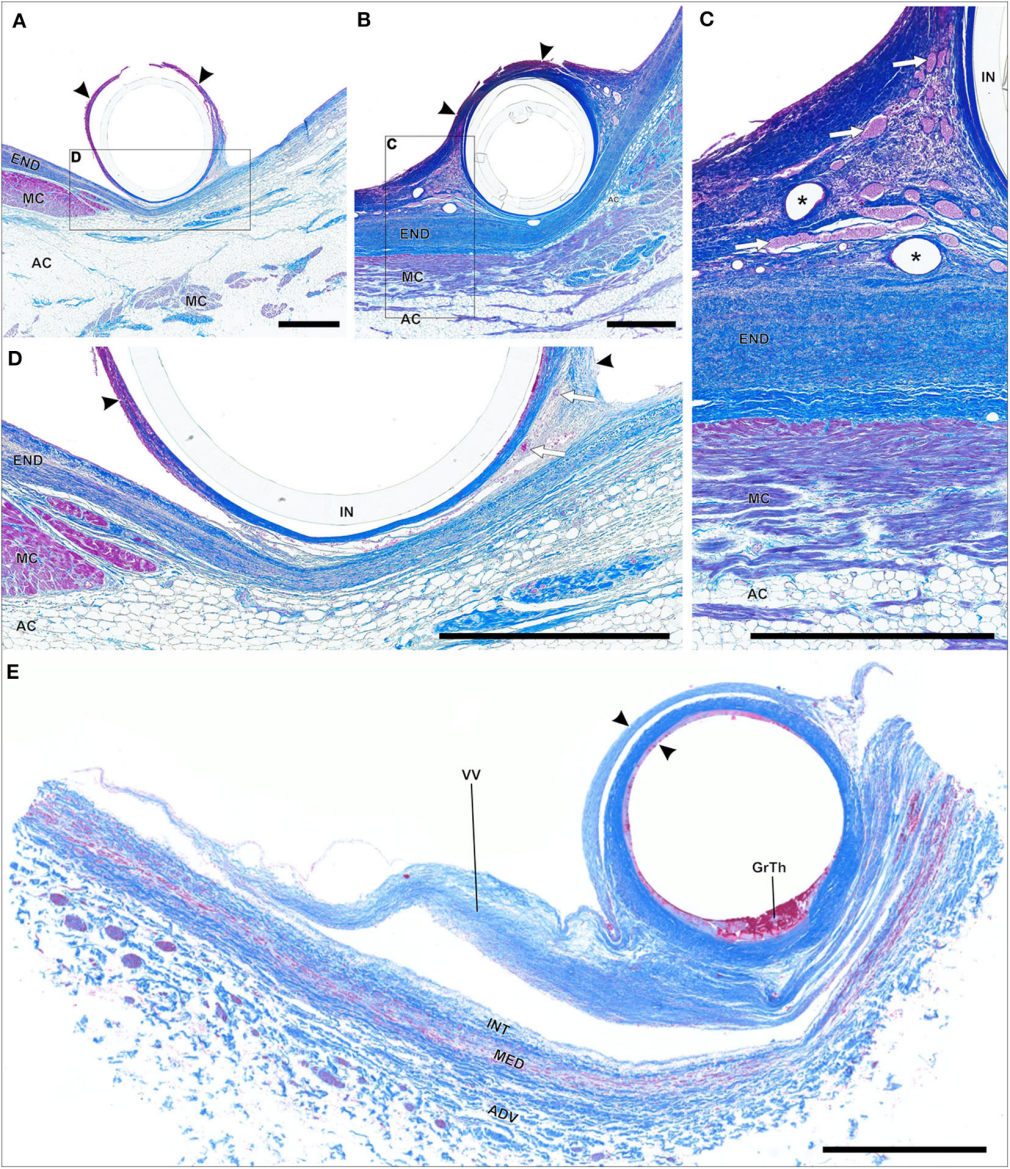
**(A–E)** Paraffin sections of thrombo-fibrotic lead encapsulations (TFLEs), stained with Azan trichrome, representing wall binding (WB) of single leads. Depicted sections represent various degrees of TFLE, dwelling times (DT) and anatomic locations. The inner metallic lead wires were removed before sectioning. **(A,D)** TFLE (arrowheads) of right ventricular (RV) lead (DT: 15 months) at the cavoatrial junction (CAJ) with dense collagenous fibers adjacent to the cardiac tissue and granular-thrombotic tissue in the distant portion with artificial rupture. Adhesion between TFLE and cardiac tissue is rather loose with moderate vascularization (arrows). **(B,C)** TFLE (arrowheads) of atrial lead (DT: 15 months; same body donor as in **A,D**) at the CAJ with pronounced vascularization (arrows) in the lateral pendentives. Blood cells in some larger vessel are rinsed (asterisks). **(E)** Double-walled TFLE (arrowhead) of RV lead (unknown DT) adhered to a venous valve (VV) at the upper caval vein bifurcation. In the inner TFLE layer granular-thrombotic tissue (GrTh) present. AC, adipose cells; ADV, adventitia; END, endocardium; IN, remained lead insulation; INT, intima; MC, myocardium; MED, media. Scale bars = 1 mm.

**Figure 10:**
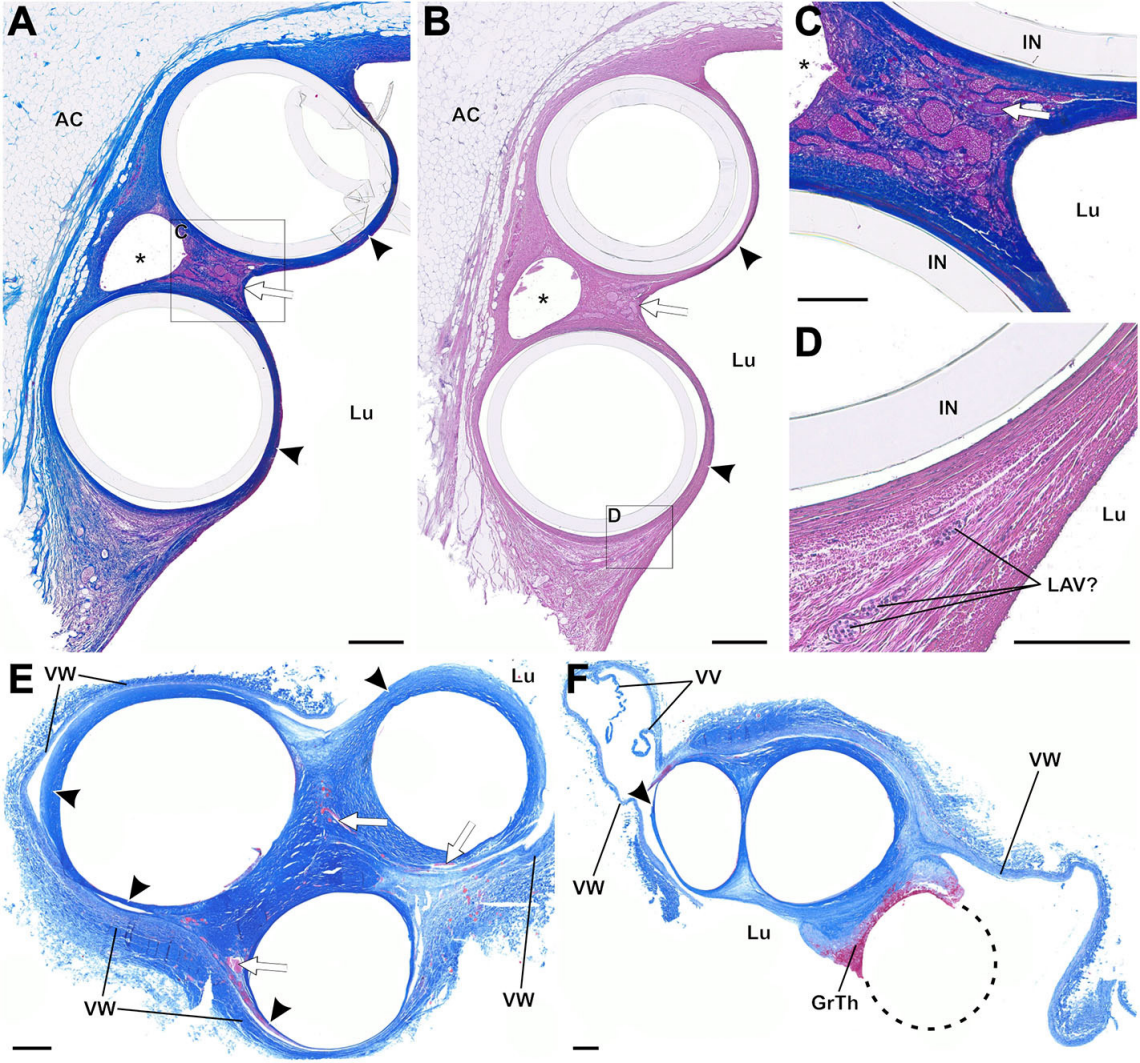
**(A–F)** Paraffin sections of thrombo-fibrotic lead encapsulations (TFLEs) with wall-binding (WB) and lead-to-lead binding (LLB), stained with Azan trichrome **(A,C,E,F)** and hematoxylin-eosin **(B,D)**. Depicted sections represent various dwelling times (DT) and anatomic locations. The inner metallic lead wires were removed before sectioning. **(A–D)** TFLE (arrowheads) of right atrial (RA) and right ventricular (RV) lead (DT: 15 months) at the cavoatrial junction (CAJ; same leads as depicted in Figures 9A,B). The dense collagenous fibers are highly vascularized in the ridge of the LLB (arrow in **A,B**). A single arteriole (arrow in **C**) runs between the marked venous plexus in the LLB. In one lateral pendentive (magnified in **D**) a presumably leukocyte associated vascularization (LAV) is visible. **(E,F)** Triple LLB of RA, RV and left ventricular (LV) lead in the brachiocephalic vein (DT: 28 months). The TFLEs (arrowheads) in **(E)** are composed of dense collagen fibers with various adhesion to the adjacent venous wall (VW). Vascularization is moderately pronounced (arrows in **E**). In a further distal portion of the TFLE **(F)**, the RA lead (dashed circle) was adhered to the adjacent RV lead without full encapsulation. AC, adipose cells; GrTh, granular-thrombotic tissue; IN, remained lead insulation; Lu, vessel lumen; VV, venous valve cusps; VW, venous wall. Scale bars = 500 μm **(A,B,E,F)** and 200 μm **(C,D)**.

In most cases, the exemplarily examined TFLEs consisted of multiple tissue components (score #2) each representing different stages of thrombus organization and fibrotic reaction (Figures 7, 8). Early fibrotization through deposition through collagen fibers into the TFLE was observed as early as 2 weeks after implantation (Figure 7). The majority (93.6%) of the histologically studied adhesive TFLEs (i.e., WB; *n* = 78) consisted of mainly collagenous fibers (score #3) with only minor portions of granular-thrombotic tissue. In contrast, composition of non-adhesive TFLEs (i.e., FRS; *n* = 26) was rather variable with either granular-thrombotic tissue (28.0%), densely packed collagen fiebers (44.0%) or mixed tissue (28.0%). TFLE wall thickness varied along the individual cross sections (Table 4). The “pendentive” lateral to encapsulated lead and the medial ridge in a lead-to-lead-binding were often highly vascularized by larger vessels (Figures 9, 10).

**Table 4:** Maximum thickness of adhesive thrombo-fibrotic lead encapsulations (TFLE with wall binding/WB) at different anatomical loci for 21 exemplary leads.

Mean (max.) WB thickness (*n* = 102)	416.3 ± 376.3 μm
Maximum WB thickness @ TCV (*n* = 7)	2155 μm
Maximum WB thickness @ CAJ (*n* = 15)	977 μm
Maximum WB thickness @ SCV (*n* = 20)	496 μm
Maximum WB thickness @ BCV (*n* = 21)	878 μm

*BCV, brachiocephalic vein; CAJ, cavoatrial junction; SCV, superior caval vein; TCV, tricuspid valve*.

#### Scanning Electron Microscopy (SEM)

**Figure 11:**
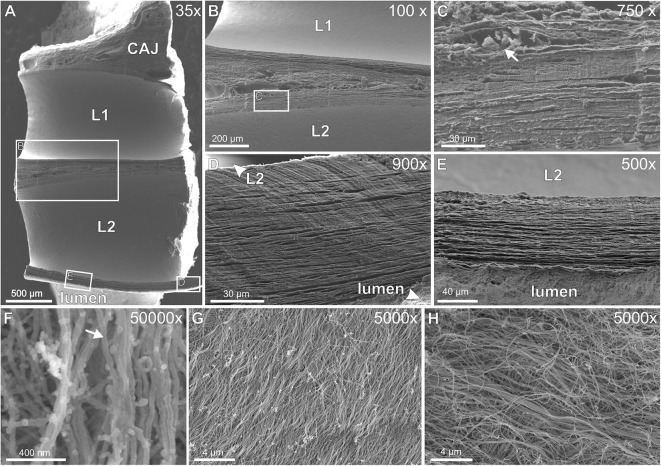
**(A–H)** Scanning electron microscopy of decellularized thrombo-fibrotic lead encapsulations (TFLEs). **(A)** Horizontal cut of a lead-to-lead-binding at the cavoatrial junction. The leads were removed before sample preparation. **(B)** Magnification of the cut face of medial ridge in **(A)**. **(C)** Magnified area in **(B)** showing both densely packed fiber collagenous mats and interjacent blood vessels (arrow) with remnants of erythrocytes. **(D,E)** Magnification of the cut face of the outer ridge between lead and vessel lumen. Parallel fiber mats were packed some more densely near lead side. **(F)** Single collagenous fibers (arrow) at strong magnification. **(G)** Lead-sided inner layer of TFLE at a right ventricular trabecula. **(H)** Subendothelial outer layer of TFLE at the tricuspid valve. Dwell time: 15 **(A–G)** and 61 months **(H)**. CAJ, cavoatrial junction; L1/L2, lead cavities.

SEM of decellularized TFLEs at the cavoatrial junction revealed the collagenous fiber texture of the subendothelial neointima, the lead-TFLE-interface and fiber arrangement in TFLE cross sections (Figure 11). Exposed single subendothelial collagenous fibers possessed a diameter of about 40 nm. A distinct correlation between lead and fiber orientation could not be seen. The lead-sided portions exhibited parallel—to some extent strongly—densely packed collagenous fiber mats which correlates with the histological findings.

## Discussion

Adhesion caused by thrombo-fibrotic lead encapsulations (TFLE) is a serious secondary complication in cardiac arrhythmia treatment based on implantable electronic devices with transvenous leads (TVL) (37–39).

### TFLE Characteristics

In agreement with previous observations (20, 24) the tissue composition of TFLEs in our samples was mainly mixed (thrombic + collagenous fibers) and focal accumulations of inflammatory cells in our samples were scarcely observed. Our examination with SEM in the sub-micron range has shown that the dense collagenous TFLEs consist of fiber “mats” parallel to the lead body and, according to previous works, consist predominantly of collagen type I (24).

The TFLE thickness in our samples of several hundred micrometers was similar to that measured in other studies (24). Contrary to previous suggestions (24), however, interindividual variations in thickness are most probably not only due to different extraction techniques during transvenous lead extraction (TLE) but mainly reflect “naturally grown” intra- and interindividual variations of TFLEs. Generally, reliable quantification of TFLE tissue characteristics [e.g., of the portion of myofibroblasts, see (24)] after TLE or *post mortem* is problematic due to the highly variable technical and biological properties of leads and patient, respectively, and because of the varying tissue morphology along a single lead.

Since tissue ingrowth in the outer insulation of the lead body is not observed in current leads it has been suggested that the high friction encountered during TLE is based mainly because of TFLE contractions mediated by myofibroblasts while involvement of vascular smooth muscle cells has been excluded (24). This assumption might be partially correct in that myofibroblast contraction contribute to some degree to the adhesive properties of the TFLE itself. We assume, however, that the individual total TFLE length of considerable 150 mm on average is the prevailing factor for high traction resistance in TLE. An effect due to different surface modifications of the ring electrode (anode) could not be seen in our study. The same is true for the insulation material of the lead body and lead diameter.

### TFLE Formation

As a rather benign concomitant after TVL implantation, TFLEs emerge presumably after a thrombotic event caused by lead-induced endothelial rupture (25, 33) or thrombus formation due to altered hemodynamics caused by lead body or lead surface. Free sheaths (i.e., TFLEs without adhesion to adjacent tissue) are probably either formed *de novo* by thrombotic accretion or grow by successive accretion at the end of an existing adhesive TFLE.

Moderate to strong foreign body reactions which are common after implantations (40) can occur at the vessel-lead interface (34) potentially impeding transmural electric impulse transfer by fibrotization of subendocardial myocardial tissue and thus increasing excitation thresholds (41, 42).

Similar to the foreign body reaction at the lead-vessel interface leucocytes/monocytes/macrophages infiltrate the freshly formed thrombus coating the lead at its luminal surface (25) contributing to thrombus organization and remodeling (35).

Steroid releasing systems adjacent to or integrated in the lead electrodes have widely proven to reduce pacing thresholds early after implantation due to their anti-inflammatory effect preventing the formation of fibrotic tissue with adverse conduction properties (43). As shown previously, these anti-fibrotic effects are not generally linked with a reduction of TFLE (21). Similarly, we observed distinctly ingrown lead-tips despite a steroid-releasing system (although our study lacks an adequate control group). These findings could be mis-interpreted as a result of the long-term depletion of the steroid depot. We observed two leads with an ensheathed tip and a dwelling time of only 2 weeks. Anti-inflammatory corticosteroids do not protect against mechanical rupture of the vascular endothelium and a possible resultant thrombus formation indicating the possible start of a TFLE. Quite the contrary, there are indication that corticosteroids could even promote thrombosis (44) possibly facilitating TFLE formation.

### TFLE Calcifications

Although veins are generally not prone to mineralization—with rare exceptions (45)—we found occasional moderate to strong calcifications in our samples, especially in the upper venous portions. The observed calcification were located rather in the TFLE than in the proper vein wall and indicate the predisposition for chronic local inflammatory processes (46) during TFLE formation. Calcifications along the leads were also seen in previous studies [e.g., (21, 26)]. This kind of TFLE induration constitutes an additional challenge during TLE and may necessitate a customized proceeding such as the successive combination of a laser and a rotating mechanical sheath (47). Medical data for our study population were underreported to conclude a correlation between certain disorders and TFLE characteristics such as the degree of calcification. Suggested risk factors for TFLE calcification are young patient age and long lead-dwelling time (48). Chronic renal disease seems to additionally promote calcium incorporation in TFLEs (26). Factors associated with arterial calcification such as uremia and diabetes mellitus (49) have been suggested to likewise predispose veins for mineralization (25) while diabetes in turn was suggested to be a predictive factor for a lower risk for lead-related venous stenosis and occlusion (50).

### Future Perspectives

Despite ongoing progress in enhancing lead design reducing technical defects such as fractures and insulation defects, TLE is necessary for an infinitive period, particularly due to lead-related infections. As a consequence, excessive TFLE persist as a serious challenge interfering with TLE, causing higher costs (51) and periprocedural risks (39), e.g., for vein injury (38). However, we actually assent to the view that moderate TFLE adhesions are desirable for leads due to a certain mechanical stability after implantation (24). Therefore, the combination of a broad suppression of TFLE formation along the lead and a pointedly and delimited stimulation for TFLE growth might provide a good strategy to prevent excessive TFLE.

Leadless pacemaker systems have the advantage of eliminating the risk for lead failure and thrombo-fibrotic lead encapsulations or lead infections but they bear their own risks and limitations (52, 53).

### Strengths and Limitations of the Study

Our study has strengths and limitations. One advantage of our study is the combination of macroscopic topography and structural analysis via histology and SEM which allowed a comprehensive characterization of the TFLE. Although not statistically powered due to the limited sample size given the various individual ages, morbidities and dwelling times, our data impart novel knowledge in TFLE parameters which should be relevant for optimizations in CIED lead design and TLE. In clinics, examination after TLE is usually restricted to strongly ruptured TFLE remnants. Studies with animal model have ethical and time limitations. Examination of body donor tissue allows the detailed study of TFLEs *in toto* and of a broad range of dwelling times.

Shrinkage due to the fixation process and conservation in ethanol may have occurred equally in all vascular tissues, although inconsistent fixation might have happened in some body donors. Histology and SEM were done only exemplarily, although intraindividual variation along the TFLEs were apparent. Complete structural screening, however, was not feasible due to the extended TFLE lengths (150 mm overall mean length per patient). The exemplary samples, however, give a good image of the histomorphological spectrum in TFLEs.

Conclusive statistics on the effect of technical lead parameter such as insulation material, surface modifications, or lead diameter on the degree of TFLE demand experimental studies.

### Conclusions

In the light of current limitations of leadless pacemakers, CIEDs equipped with electrode and shock coil leads will remain the means of choice for the next decade or longer. *Post mortem* analysis of CIED leads helps to understand both characteristics and formation of lead-induced thrombo-fibrotic encapsulations. The combination of a selective facilitation of lead ingrowth by surface-modifications and a, e.g., drug-based suppression of ingrowth might improve extractability due to a reduced overall ingrowth in case of infection or lead dysfunction.

## Data Availability Statement

The raw data supporting the conclusions of this article will be made available by the authors, without undue reservation.

## Ethics Statement

The study was reviewed and approved by the ethics committee of the Rostock University Medical Center (vote A2015-0141).

## Author Contributions

JK, MS, and AW designed and planned the project. JK and MS carried out the TFLE dissections. JK, RD, and AÖ analyzed the device data. JK and AS performed SEM. JK analyzed the TFLE data and designed concepts for figures. JK, MS, AÖ, and AW interpreted the TFLE data. JK wrote the manuscript. All authors contributed to the article and approved the submitted version.

## Conflict of Interest

JK has received research grants from Biotronik. The remaining authors declare that the research was conducted in the absence of any commercial or financial relationships that could be construed as a potential conflict of interest.
